# Navigating the diagnostic and management challenges of paratubal cysts in adolescents: A case report and primary care insights

**DOI:** 10.51866/cr.956

**Published:** 2025-11-04

**Authors:** Poh Yi Ooi, Imran Ahmad, Abdul Kadir Azidah

**Affiliations:** 1 MBBS, MMed (Family Medicine), Department of Family Medicine, School of Medical Sciences, Universiti Sains Malaysia, Kubang Kerian, Kelantan, Malaysia. E-mail: profimran@usm.my; 2 MBBS, Department of Family Medicine, School of Medical Sciences, Universiti Sains Malaysia, Kubang Kerian, Kelantan, Malaysia.; 3 MD, MMed, PhD, Department of Family Medicine, School of Medical Sciences, Universiti Sains Malaysia, Kubang, Kerian, Kelantan, Malaysia.

**Keywords:** Paraovarian cyst, Abdominal pain, Adolescent

## Abstract

Abdominal pain frequently drives patients to seek care at family physician clinics and emergency departments. It often signifies serious underlying conditions, presenting diagnostic challenges for physicians. Failure to diagnose an acute abdomen can result in patient morbidity and mortality and medicolegal risks for physicians. Herein, we report an unusual gynaecological case involving a paratubal cyst in a 15-year-old girl who presented with acute abdominal pain. This case underscores the critical role of primary care physicians in identifying red flags and initiating timely specialist referral. Abdominal ultrasound revealed a large, unilocular mass in the right adnexa, measuring 18x9.8 cm. The patient underwent an emergency laparotomy with a clinical diagnosis of a twisted ovarian cyst. However, intraoperative findings revealed a right paratubal cyst measuring 18x16x16 cm, which was not twisted. A fertility-preserving cystectomy was subsequently performed, successfully removing the cyst while preserving the ovary. Histopathological examination confirmed it as a benign paratubal cyst, consistent with the intraoperative findings. Although paratubal cysts are uncommon, they are a significant cause of abdominal pain. Therefore, timely identification, thorough clinical evaluation and appropriate diagnostic tests are essential for optimal patient care.

## Introduction

Abdominal pain is a common and challenging primary care complaint, with a reported consultation rate of 2.8% in Western countries.^1^ While Malaysian data may differ, its wide range of differential diagnoses—from nonspecific causes (20%) and gastroenteritis (13%) to appendicitis (12%) and gynaecological conditions (9%)—makes accurate assessment crucial.^2^ Approximately 25% of cases presenting with abdominal pain in primary care require urgent hospital referral.^2^ Therefore, accurate history-taking and physical examination are essential to avoid misdiagnosis and treatment delays.

Paratubal cysts, also known as paraovarian cysts, are fluid-filled sacs located adjacent to the ovary and fallopian tube, typically within the broad ligament of the uterus.^3^ Paratubal cysts are most prevalent during the third and fourth decades of life, with only 4% occurring in the adolescent population.^4^ The average paratubal cyst is 7.51 cm, with only 12.9% exceeding 10 cm and thoese over 15 cm defined as “giant”.^3,5^ Reports of such giant cysts in adolescents are rare.^5^ Here, we present the case of a 15-year-old girl with abdominal pain caused by a right giant paratubal cyst, which was successfully managed following a prompt gynaecology referral.

## Case presentation

A 15-year-old girl presented to a primary care clinic with acute-onset right lower abdominal pain for a day. The pain was described as intermittent and sharp, with a severity of 8/10. Examination revealed a palpable abdominal mass corresponding to a 20-week gravid uterus, prompting an urgent referral to the gynaecology department for evaluation. Upon hospital arrival, further history taking noted progressive abdominal distension over the past year, which she had attributed to weight gain, along with irregular menstrual cycles. She denied fever, vomiting or urinary symptoms. She was nulliparous, sexually inactive, and had a maternal history of uterine fibroids. Vital signs were normal. Abdominal palpation revealed a large, tender mass in the right lower quadrant. Her pain was managed with intravenous tramadol 50 mg, which reduced her pain score from 8/10 to 1-2/10.

Urine pregnancy test was negative, while routine blood tests and urinalysis were normal. Tumour markers were also within normal limits: alpha-fetoprotein (AFP) level of 2.03 IU/mL, carbohydrate antigen 19-9 (CA19-9) level of 12.7 U/mL and carcinoembryonic antigen (CEA) level of 3 μ/L.

**Figure 1:**
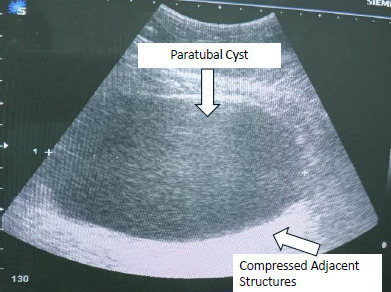
Abdominal ultrasound demonstrating a large right-sided unilocular adnexal mass, later confirmed to be a paratubal cyst, measuring 18 cm in its largest diameter.

Abdominal ultrasound revealed a large rightsided unilocular adnexal mass measuring 18×9.8 cm, displacing the uterus posteriorly. The uterus measured 6×3.5 cm, with an endometrial thickness of 6.7 mm. There were no free fluid, solid components or papillary projections (Figure 1).

**Figure 2:**
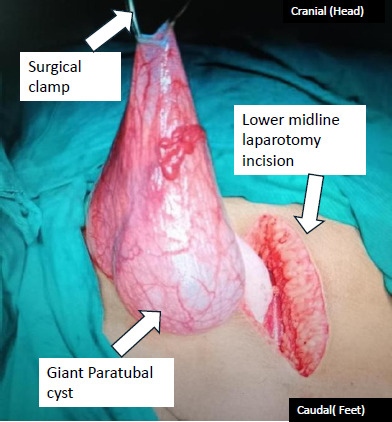
Intraoperative view of the 18×16×16- cm right paratubal cyst after being exteriorised from the abdomen.

Based on a clinical diagnosis of a twisted right ovarian cyst, an emergency laparotomy for cystectomy with possible unilateral salpingo-oophorectomy was planned. Intraoperatively, a right paratubal cyst measuring 18x16x16 cm was identified (Figure 2). The cyst was not twisted but was adherent to the right ovary, which appeared overstretched but otherwise healthy. The left ovary, fallopian tube, uterus, and appendix were grossly normal, with no free fluid or endometriotic lesions in the pouch of Douglas.

A midline laparotomy and cystectomy were performed. After decompressing 2000 cc of straw-coloured fluid, the cyst wall was excised. Histopathological examination subsequently confirmed a benign paratubal cyst.

**Figure 3:**
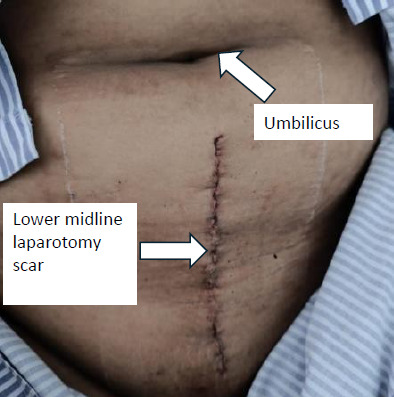
Surgical wound on postoperative day 2.

She was discharged on postoperative day 2 without complications (Figure 3). At her 1-month follow-up, she was well with no complaints.

## Discussion

**Figure 4:**
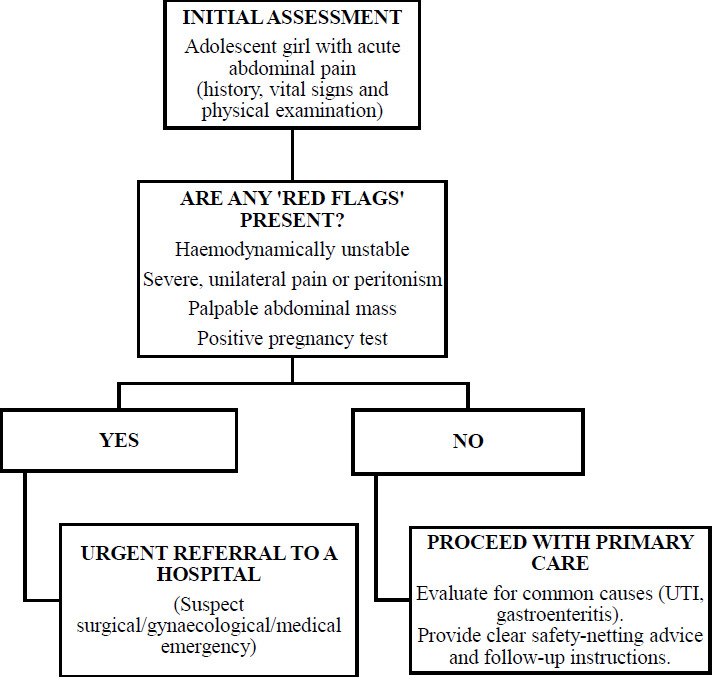
Primary care approach to acute abdominal pain in an adolescent girl.

The initial presentation exemplifies a diagnostic challenge in primary care setting. Acute abdominal pain in adolescent girls requires a rapid, systematic evaluation, as the differential diagnosis is broad which including emergencies like ectopic pregnancy and ovarian torsion (Table 1). The decision of urgent referral was warranted based on a clear constellation of ‘red flags’. Specifically, the combination of 1) acute-onset, 2) severe, unilateral abdominal pain and 3) a large, palpable abdominal mass indicates a surgical emergency. Recognising this and prioritising immediate referral over further primary care investigations were the top priorities. A proposed diagnostic algorithm is presented in Figure 4.

**Table 1:** Differential diagnosis of a large adnexal mass in an adolescent.^6^

Category	Diagnosis	Key clinical features
Gynaecological (benign)	Functional ovarian cyst	Asymptomatic/mild pain;resolves spontaneously
	Paratubal/paraovarian cyst	Asymptomatic unless large; causes pressure/torsion
	Ovarian torsion	Acute, severe, unilateral pelvic pain; often with nausea/ vomiting; surgical emergency
	Endometrioma (‘chocolate cyst’)	Chronic pelvic pain, dysmenorrhoea; associated with infertility
	Tubo-ovarian abscess	Fever, pelvic pain, cervical motion tenderness, vaginal discharge
	Ectopic pregnancy	Amenorrhoea, abdominal pain, vaginal bleeding; positive pregnancy test
Gynaecological (malignant)	Germ cell tumour (e.g. teratoma)	Pain/mass effect; tumour markers (AFP and hCG) possible elevated
	Epithelial tumour	Abdominal distension/bloating; CA-125 level possible elevated
Non-gynaecological	Appendiceal abscess	Fever, right lower quadrant pain, nausea; often follows perforated appendicitis
	Distended bladder	Suprapubic mass, difficulty urinating/urinary retention
	Mesenteric cyst	Vague abdominal pain /asymptomatic abdominal mass

Abbreviations: AFP, alpha-fetoprotein; hCG, human chorionic gonadotropin; CA-125, cancer antigen 125

An inexperienced clinician might initially attribute the symptom of sudden-onset right lower abdominal pain to acute appendicitis. However, detailed gynaecological history-taking would reveal a year-long history of irregular menses. Furthermore, the abdominal distention, which the patient perceived as insignificant, was a crucial diagnostic clue. This highlights the importance of detailed history-taking, which yields accurate diagnosis in 75% of cases, whereas physical examination and investigations contribute 12% and 11%, respectively.^7^

Paratubal cysts account for about 10% of adnexal masses and can mimic other conditions, such as appendicitis.^4,8^ In a study of 283 adolescent girls with suspected acute appendicitis, 44 actually had incidental paratubal cysts, and two had torsion of large cysts.^8^ While usually small and asymptomatic, paratubal cysts occasionally grow large, causing abdominal pain, distension, torsion, rupture or menstrual irregularities.^5^ Giant paratubal cysts (>15 cm) are particularly uncommon in adolescents with only 27 giant paratubal cyst cases globally, 10 in patients under 18 years old.^9^ To our knowledge, this is one of the few such cases documented in Malaysia, highlighting its regional significance.

Differentiating paratubal from ovarian cysts on imaging is challenging; they are often misdiagnosed as ovarian cysts (preoperative suspicion in only about 1 in 15 patients).^3^ This difficulty stems from identifying the ‘split sign’, which involves distinguishing the cyst from the ipsilateral ovary by applying pressure with the ultrasound transducer, especially when the cyst is large or multilocular.^3^ This was reflected in our case, where the preoperative diagnosis was a twisted ovarian cyst. Assessing adnexal masses for malignancy is essential, as the risk increases with cysts larger than 10 cm.^6^ While sonographically, benign paratubal cysts appear as unilocular with thin walls and smooth margins, features like multiloculation, solid components, thick septations, nodularity, papillary projections or Doppler-detected neovascularisation suggest malignancy.^10^ CT or MRI may aid in uncertain cases, given the acute clinical presentation and the reassuring ultrasound features, the decision for immediate surgery was made. While other tumour markers (AFP, CA19 9 and CEA) were obtained, CA 125 testing, an epithelial ovarian tumour markers was not ordered. Ideally, CA 125 should have been included, but in this emergency setting, the priority was to expedite surgical intervention to prevent possible ovarian loss. This omission represents a limitation of the preoperative workup.

Surgical management of paratubal cysts involves choosing between laparoscopy and laparotomy based on cyst size, malignancy suspicion, and surgeon’s expertise. Laparoscopy is increasingly favoured due to reduced postoperative pain, faster recovery, and superior cosmesis.^11^ Bhansakarya and Subedi described a 20x18-cm cyst managed laparoscopically after drainage with a Veress needle after a low-risk malignancy index assessment.^12^ In our case, the CA-125 level was not measured, thus preventing the determination of the malignancy risk index. Also, laparoscopy presents technical challenges with giant masses within limited intrabdominal space. Conversely, laparotomy provides direct access and excellent exposure, allowing for controlled removal with a lower risk of spillage and thorough inspection.^10^ Our decision for laparotomy stemmed from the cyst’s size, acute presentation and inability to rule out malignancy preoperatively, aiming for safe ovary-preserving excision. The patient’s mother too consented to an open procedure, aligning with the surgical team’s recommendation.

Fertility preservation is the priority when managing adnexal masses in adolescents. While the primary treatment is cystectomy, giant cysts may require tubal excision or oophorectomy. A recent review supports an ovarian-sparing surgery approach, reporting that 85.1% of paratubal cyst surgeries preserved the ovary, with a low torsion risk of 11.1%.^9^

Benign paratubal cysts have a low recurrence risk after complete excision.^4^ Follow up includes annual check-ups and a pelvic ultrasound considered if symptoms like abdominal pain or menstrual changes recur.

Beyond physical considerations, the psychological impact of diagnosis and surgery on adolescent’s body image and wellbeing requires attention. Hence, preoperative counselling and postoperative emotional support are crucial.

## Conclusion

The successful outcome in our patient underscores the importance of including paratubal cysts in the differential diagnosis of adolescent adnexal masses. For primary care physicians, this serves as a reminder to recognise red-flag symptoms in adolescents with abdominal pain to facilitate timely, life-preserving interventions and to provide holistic, compassionate care.
